# A multi-level model of emerging technology: An empirical study of the evolution of biotechnology from 1976 to 2003

**DOI:** 10.1371/journal.pone.0197024

**Published:** 2018-05-24

**Authors:** Ad van den Oord, Arjen van Witteloostuijn

**Affiliations:** 1 Innovation Lab, Eindhoven University of Technology, Eindhoven, the Netherlands; 2 Jheronimus Academy of Data Science, ‘s-Hertogenbosch, the Netherlands; 3 Tilburg School of Governance / Economics and Management, Tilburg University, Tilburg, the Netherlands; 4 Antwerp Management School / University of Antwerp, Antwerp, Belgium; Institut Català de Paleoecologia Humana i Evolució Social (IPHES), SPAIN

## Abstract

In this paper, we develop an ecological, multi-level model that can be used to study the evolution of emerging technology. More specifically, by defining technology as a system composed of a set of interacting components, we can build upon the argument of multi-level density dependence from organizational ecology to develop a distribution-independent model of technological evolution. This allows us to distinguish between different stages of component development, which provides more insight into the emergence of stable component configurations, or dominant designs. We validate our hypotheses in the biotechnology industry by using patent data from the USPTO from 1976 to 2003.

## Introduction

In our industrial and post-industrial societies, technology plays a highly important role, structuring the relationship between individuals, groups, organizations, and industries [1, 2, 3, 4]. Emerging technologies–such as, for example, biotechnology and nanotechnology–are considered especially important, as these are argued to be a key driver of economic growth and development [5]. Unfortunately, however, the process of how emerging technologies grow and evolve is not well-understood [6]. While scholars have devoted much time and effort to understand the evolution of mature (i.e., non-emerging) technologies, insights into the evolution of emerging technologies have severely lagged behind. The main reason is that mature technologies are characterized by stable and predictable patterns of growth, whereas emerging technologies are associated with highly chaotic and fluid patterns of growth and development [7, 8, 9]. This implies that a mature technology can be modeled relatively easily, whereas emerging technologies defy traditional means of modeling.

In this paper, we argue that by considering the multi-level nature of technology, we can generate valuable insights into the growth and evolution of emerging technologies. As technology is inherently multi-level in nature [10], we conceive technology as comprising a system composed of a set of interacting technological components. This allows us to study an emerging technology on the basis of the growth and evolution of its principal components [11]. Specifically, we bring in insights from organizational ecology. We use organizational ecology's multi-level density dependence theory to study component-system interactions [12], and define different stages of technological development at the component level, as well as at the system level. We empirically validate our hypotheses in the biotechnology industry from 1976 to 2003 by using patent data from the United States Patent and Trademark Office (USPTO).

The contribution of this paper is threefold. First, by conceiving technology as a system that is composed of a set of interacting technological components, we develop a multi-level model that can be used to study the evolution of (emerging) technology. This allows us to examine the microeconomic underpinnings of an emerging technology’s growth and development, and explain how stable patterns of growth and development originate from the bottom up. Second, by applying concepts from organizational ecology to the study of technology, we effectively combine evolutionary and ecological models in the study of the evolution of technology (cf. [13]). That is, by combining the different perspectives of environmental selection and adaptation, we demonstrate the added value of applying both views at the same time, and provide a platform for their further recombination (cf. [14]). Third, we empirically demonstrate that an ecological logic can be effectively applied to study the growth and evolution of emerging technology.

The organization of this paper is as follows. In the next section, we will discuss the literature on the evolution and, on the basis of these insights, develop our hypotheses. Subsequently, in the ‘Data and methodology’ section, we outline our empirical sample and estimation methods. We then discuss our results, and finally, in the ‘Discussion and conclusion’, we discuss the limitations of our study, position our findings in the broader academic debate, and provide some thoughts on avenues for further research.

## The evolution of technology

Even though economists already recognized the importance of technological progress early on [15], they generally captured technological change as a mere shift along the production function. As a result, the process of technological change has largely remained unexplored until Schumpeter [16] presented an evolutionary theory of the working of the capitalist system, driven by forces of technological change. Schumpeter [17] conceived technological change as a process of recombination, where (existing) components are brought together in new ways. Since then, the process of technological change has no longer been treated as a ‘black box’ [18], and has been–and still is, for that matter–receiving much attention.

Especially the field of evolutionary economics has contributed much to our understanding of the process of technological change. According to evolutionary economics, due to an agent’s limits in information-processing and problem-solving capacity, or bounded rationality [19], there is a need to search and recombine locally from a limited set of components [20]. After all, if the number of components that an agent considers increases linearly, the number of potential (re-)combinations that can be made with these components and the associated cognitive load grow exponentially [21]. Therefore, agents rely on heuristics to reduce their cognitive load, rather than applying strict and rigid rules of optimization [19].

At the organizational level, these heuristics translate into organizational routines that ensure regular and predictable patterns of behavior [2] that result in organizational inertia [22] and path dependence. In the context of technological development, these heuristics result in stable and predictable patterns of technological progress. These heuristics are well documented in the literature, albeit under many different names. For example, Rosenberg [23] argues that technology acts as a so-called focusing device, where typical problems, opportunities and targets direct technological search (i.e., growth and development) in particular directions. According to Sahal [24], technological guideposts and basic designs lay out definite paths of development characterized by long periods of incremental improvements. In building upon Kuhn’s [25] notion of scientific paradigms, Dosi [7] posits that the characteristic habits and routines in searching and problem solving on the ground of a technological paradigm result in path dependence. In the remainder of the current paper, we will refer to the heuristics that underlie stable and predictable patterns of technological growth as stable technological design configurations.

While these stable and predictable technological design configurations lead to stable and predictable patterns of technological growth, they do not emerge ex nihilo, implying at least two stages of technological evolution. These two stages of technological evolution have also been documented extensively in the literature. For example, according to Utterback and Abernathy [26], technological development evolves from an uncoordinated process, characterized by fluid and unsettled relationships, into an efficient and tightly integrated system with highly specialized and interdependent actors. Clark [27] argues that as development proceeds, technological diversity gives way to standardization, where performance criteria and processes are more clearly specified. Furthermore, Anderson and Tushman [8] identify long eras of cumulative and incremental changes that are punctuated by concise periods of ferment or brief periods of major discontinuities.

Hence, technological growth and evolution is characterized by two stages of development. Initially, during the stage of emergence, through a process of social construction [28], stakeholders in the environment seek to find the technological design configuration that enables cumulative growth and development. Subsequently, during the stage of maturity, the stakeholders in the environment have converged toward a single (stable) technological design configuration (or, in popular parlance, a dominant design), which results in stable and predictable patterns of growth and development. This means that we define an emerging technology as a technology that is in an early stage of development [29, 30], characterized by the absence of a stable technological design configuration. Due to this absence, this stage is characterized by high uncertainty, as the exact form, capabilities, constraints, and uses of the technology are still in flux [29, 30, 31, 32]. Accordingly, we define a mature technology as a technology that is characterized by the existence of a stable technological design configuration that directs growth and development in a predictable way.

The stable and predictable patterns of technological growth that characterize mature technologies are much better suited for formal modeling than the chaotic patterns of change that are associated with emerging technologies. As we will explain below, formal models that are tailored to study the growth and evolution of mature technologies are not well-suited to study emerging technologies. Although emerging technologies are studied indirectly as part of mature technologies, this is only done in retrospect, which severely limits our understanding of the growth and evolution of emerging technology. When studying diffusion processes and population evolution prospectively (i.e., while they evolve), better and more unbiased insights result [33].

## Technology lifecycle models

In what is considered as one of the first scientific works that treats the development of new technology as an economic phenomenon, Zvi Griliches [34] found that the penetration of corn seeds follows a characteristic S-shaped growth pattern (i.e., Pearl Reed/Logistics curve). Since then, numerous empirical studies have contributed to establishing the S-shaped pattern as a general rule for technological growth and the diffusion of innovation [35, 36, 37, 38]. On the basis of this characteristic growth curve, numerous forecasting models have been developed [39], which can be used to delineate the different stages of technological evolution. Basically, this methodology implies estimating the Logistics growth curve, and then distinguishing between the different stages using threshold values of the cumulative density function of this estimated growth curve. These so-called ‘technology lifecycle’ or TLC models usually distinguish between four stages of technological evolution, namely the (1) seed, (2) growth, (3) maturity and (4) decline stage of development [40]. Table 1 provides the commonly used threshold values for the cumulative density function to distinguish between these four stages.

**Table 1:** Stages of technological evolution and threshold values of cumulative density function.

Stage of evolution	Cumulative density
Seed	0.00–0.16
Growth	0.16–0.84
Maturity	0.84–0.99
Decline	0.99–1.00

Adapted from [40].

Alternatives of the Pearl Reed/Logistics curve have also been proposed, such as an exponential [41] and Gompertz model [42]. However, all these models rely on a cumulative density function, and can only be used effectively when the upper limit of technological development is more or less known (i.e., when the cumulative density is close to its maximum value) or when the upper limit can be estimated in a reliable way–for example, on the basis of the growth patterns of highly similar technologies. This implies that these models only have a limited applicability for completely new and emerging technologies, for which the upper limit is not known and cannot be reasonably estimated. After all, due to the absence of a stable technological design configuration, the potential of emerging technologies is unknown [30, 43, 44].

Another well-known model is Bass’ [35] rate of change model. This is an empirical version of Roger’s [45] innovation diffusion model and is one of the most widely applied models in management science [46]. The reason is that this model is associated with a simple and elegant theory that explains the existence of an empirical generalization. According to Bass’ [35] model, the probability that a technology will be initially adopted is a linear function of the number of previous adopters. Bass’ model distinguishes between different stages, too, but involves the adoption of technological products, the labels describing the characteristics of the adopters in the process of technological diffusion. The labels of the different stages are as follows: innovators, early adopters, early majority, late majority, and laggards. Because Bass’ model also relies on a cumulative density function, it is also rather unstable when limited data are available [47]. This is not surprising, as Bass [35] acknowledges that the model should be supplemented with additional information (e.g., diffusion rates of comparable technologies or products).

We believe that the main reason that these models are not well-suited for studying emerging technologies is that they do not consider the multi-level nature of technology. As the phenomenon of technology is inherently multi-level in character [10], developing a systemic, multi-level model of the evolution of technology can add insights above and beyond a singular perspective. After all, a multi-level model allows for an analysis of how stable and predictable patterns emerge at lower levels of analysis, and how these travel upwards in the hierarchy of technology [48].

## A multi-level model of emerging technology

**Fig 1:**
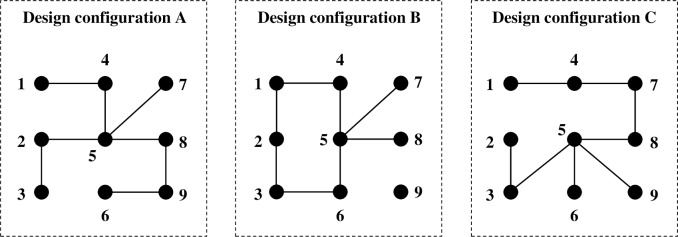
Alternative technological design configurations. The nodes are technological components.

In the current study, we develop a formal model to study the growth and evolution of emerging technology by explicitly acknowledging the hierarchical nature of technology. In nature and society, many things are organized hierarchically [49], and technology is no exception [10]. Hence, we introduce the notion of a technological system, which we define as a set of interacting technological components, embedded in the broader technological environment. On the basis of this definition, we can distinguish between emerging and mature technologies in the following way. Mature technologies are characterized by the existence of a stable technological design configuration of its main (or core) technological components, which allows stakeholders to concentrate on a single (component) configuration and stop investing in alternative configurations [50], which enables incremental and cumulative changes. Quite the opposite, emerging technologies are characterized by experimentation with different component configurations (see Fig 1), which results in highly chaotic and unstable patterns of growth and development as it is yet unclear which design configuration will be selected (i.e., become dominant) to enable cumulative and predictable patterns of growth and development.

Obviously, emerging technologies do not always display highly chaotic and unstable patterns of growth and development, as the stable design (or component) configurations that characterize mature technologies are (socially) constructed during the stage of emergence. For example, in Fig 1, some of the links between the component technologies are present in all alternative design configurations (e.g., components 1 and 4, 2 and 3, and 5 and 8 are linked in all alternative design configurations). Hence, emerging technologies can be associated with different levels of stability, and this degree of stability can actually be used as an indication of the stage of development of an emerging technology. It thus becomes important to identify stable component configurations in emerging technologies. This can be done in numerous ways.

The approach we take in the current study is to build upon insights from organizational ecology, a prominent sociological theory of the evolution of organizations and organizational populations. The reason for doing so is that the theory of density dependence is highly developed in organizational ecology. As explained above, density dependence characterizes most existing models of technological growth and development. This means that we can build upon density dependence theory from organizational ecology to build a model of emerging technologies. Organizational ecology’s density dependence theory argues that population density serves as a surrogate for the difficult-to-observe features of the material and social environment that affect population dynamics, particularly social legitimation (i.e., the standing as a taken-for-granted element in a social structure) and diffuse competition (i.e., common dependence on the same resource pool). Even though density dependence logic has been primarily applied to organizational populations [51], recent research suggests that this argument can also be effectively applied to other settings as well, such as the domain of national laws [52, 53] and organizational rules [54, 55]. In this paper, we argue that density dependence logic can also be fruitfully utilized to study technological populations, as technologies also need to be legitimized [13, 56, 57, 58, 59] and compete with one another for the attraction of resources and attention [60, 61].

Given the multi-level nature of our model, we build upon multi-level density dependence theory [12]. This theory posits that legitimation processes operate more broadly than competition, because legitimation processes are tied to information while competition operates through the flow of material resources [62]. As a result, competition is bound by physical and political boundaries, while legitimation is not (or less so). Adopting this logic to our setting, we can argue that the more integrated a technological system is, the more that its components are legitimated at the system level. Hence, by investigating the extent to which individual components are legitimated at the system level (i.e., by examining the effect of system density on component growth), we can study the evolution of emerging technology. The logic is straightforward. The more components are legitimated at the system level (i.e., the more that the growth and evolution of individual components is dependent upon the density of the technological system as a whole), the more stable the component configuration of the technological system, and the closer an emerging technology is to maturity. We acknowledge that our approach is somewhat simplistic, as it assumes the existence of only one stable component configuration. Additional insights can be generated when allowing for the existence of multiple configurations (e.g., by looking at their interactions). However, we limit our discussion to the simpler case for two reasons. First, mature technologies are characterized by a single, dominant component configuration, which makes this model appropriate for our current purposes. Second, and related to the previous point, more complex models would contribute little to the main thrust of this paper, while greatly increasing the complexity of our theory and analyses.

The component-system interaction of an emerging technology (i.e., the legitimation of technological components at the system level) also implies (at least) two stages of development at the component level. Initially, when a focal component is first discovered, it is unclear how this component relates to the other components (if any) of the system, implying that the component is not legitimated at the system level. In line with the different stages of the technology life cycle (see Table 1), we refer to this as the seed stage of component development. Then, as stakeholders recombine the focal component with the other components, its role in the system becomes apparent, and the component is legitimated at the system level (i.e., there is a positive interaction between the focal component and the system's remaining components). Now, the component forms an integral part of the technological system, and legitimation processes at the system level start to contribute positively to component growth. We refer to this stage as the growth stage of component development. This effectively allows us to study the evolution of emerging technologies by investigating the evolution of their principal components. Accordingly, we develop several hypotheses regarding component evolution next.

### Component density

As mentioned, we will use the effect of the density of the technological system (i.e., the aggregate density of all its components) to distinguish between two stages of component growth (i.e., the seed and growth stage). As such, we cannot develop any hypotheses about the effect of system density on the development and evolution of its component technologies. Hence, our first hypothesis explores the effect of the density of the component on its own development and evolution. For this hypothesis, we will also build on organizational ecology’s density dependence theory, by considering the technological components as populations of related technological inventions. To clarify, let us illustrate this with an example. Imagine automobile technology as the technological system. This technological system is comprised of several technological components, such as the engine, drive train, brake system, wheels, cooling system, body, et cetera. Each of these component technologies were not invented out of nothing, but are actually comprised of populations of related inventions, as scientists and engineers built upon inventions from their predecessors to develop our modern-day internal combustion engine.

As said, density dependence theory argues that population density can be used as a surrogate for the difficult-to-observe features of the material and social environment that affect population dynamics; i.e., social legitimation and diffuse competition. The theory also posits that legitimation is mainly important in the formative stages of population development, while competition is mainly important in the mature stage of population development [21, 51]. As the defining characteristic of an emerging technology is the fact that it is not mature, we do not expect to find any evidence for processes of competition, in either stage of component development. Hence, we argue that only the process of legitimation characterize the development and evolution of an emerging technology’s components, which implies that we only expect to find a positive effect of component density on its subsequent development and evolution. Regarding the process of legitimation during the different stages of component evolution, because the process of legitimation is mainly important during the early stages of population development, we expect to find a stronger effect during the seed stage than during the growth stage of component development. However, because technological growth only really takes off after a stable technological design configuration has been established, the growth of emerging technologies and their component technologies is limited. This means that we cannot be sure to find a significant difference in the effect of component density on its evolution; we can only be certain that the effect of component density is not significantly lower in the seed stage of component development. We formulate our first hypothesis accordingly as:

**Hypothesis 1:** Component density is positively associated with component development, and is not significantly lower in the growth stage of development versus the seed stage of component development.

### Organizational density

Nowadays, most technology is developed in the context of organizations. This means that organizational density plays a highly important role in the development of technology, and that the legitimation of technology is tied to the number of organizations adopting the technology [35, 63, 64]. This is the result of a process called mimetic isomorphism, where stakeholders imitate each other’s behavior under conditions of high uncertainty [65]. This process is mainly important in the seed stage of the component’s development, when the component’s role in the system is unknown, objective performance criteria are absent, and development is characterized by extreme levels of uncertainty. Stakeholders thus have to resort to alternative means to determine whether the component is worthy of their resources and attention, and start copying one another’s behavior. So, especially in the seed stage of development, due to the high levels of uncertainty [6], organizational density has a legitimating effect on technology and, therefore, a positive effect on component growth. In the growth stage of development, the legitimation of technology remains important, and technological developments are still characterized by high levels of uncertainty. However, as the role of the component in the system becomes more apparent, uncertainty is significantly lowered, and legitimation processes start to operate more and more at the system level. Therefore, we expect legitimation effects of organizational density to be less strong in the growth stage of development. This gives:

**Hypothesis 2:** Organizational density is positively associated with component growth, and more strongly so in the seed stage of development.

### Technological lineage and diversity

Conceiving technological change as a process of recombination naturally implies technological lineage, where a focal invention builds upon antecedent inventions, and can subsequently become the basis for future descendant inventions [66, 67]. This logic not only relates to individual inventions, but can also be applied at higher levels of aggregation. So, besides the focal technology of the component (i.e., the set of inventions that belong to the component), we can also identify the antecedent technology of a components (i.e., the set of inventions on which the component builds, or its knowledge base), and the descendant technology of a component (i.e., the set of inventions that build upon the component's technology, or the component's application domain). Clearly, different measures can be conceived to characterize the antecedent, focal, and descendant technology of a component. Here, we will investigate the role of technological diversity. After all, in the evolutionary logic of technological change, diversity is a central notion, forming the input to the process of recombination, therefore being considered to be the ultimate source of novelty [68, 69, 70]. Diversity is a central notion in organizational ecology as well, since initial research in the field was centered on seeking an answer to the question “Why are there so many different kinds of organizations?” [71].

### Focal component diversity

Component diversity represents the degree to which technological development within the component takes place within different subcomponents. This has different implications in different stages of technological development. On the one hand, in the seed stage of component development, more subcomponents imply more alternative subcomponent configurations, which is associated with more flexibility that prevents lock-in or path dependence [72, 73]. In this respect, component diversity represents the broadness or niche width of a technological component. The broader the appeal of the component to stakeholders, the more the component is able to mobilize resources from these actors in different parts of the technological environment, which stimulates component growth. Moreover, increasing the number of subcomponents also increases the potential for their recombination, so increasing the technological opportunities within this component and the potential value of the component in the larger technological system.

On the other hand, in the growth stage of development, the potential appeal of the component’s technology to stakeholders in the wider technological environment is no longer relevant. After all, the component’s stakeholders have collectively agreed upon working on the dominant subcomponent configuration, specified in the component’s dominant design. This enables actor specialization and facilitates cumulative development, thus transforming the component’s potential value into real techno-economic value. In doing so, a (broad) direction of technological development for the component has been chosen, and alternative directions (i.e., alternative subcomponent configurations) have been foreclosed. Hence, diversity (i.e., divergence) implies a fragmentation of resources, and hampers convergence towards the component’s stable subcomponent configuration. This suggests:

**Hypothesis 3:** Component diversity is positively (negatively) associated with component growth in the seed (growth) stage of development.

### Antecedent diversity

The antecedent technology of a component can be seen as the knowledge base on which the component builds. Because the development of the technological component is to a large extent dependent on the underlying knowledge base [64], the more diverse this knowledge base, the higher the recombination potential of the technological component [74]. Antecedent diversity yields a potential for novel combinations to emerge, implying the expectation that antecedent diversity positively impacts subsequent component growth. Although the number of possible combinations can literally grow to infinity, given the limited number of potential components that an inventor can simultaneously consider [20], it will become increasingly difficult for the involved actors to develop a sensible interpretation of all the potential novel combinations [74]. Since every component can be incorporated in further re-combinations, an actor’s combinatory potential [75] and associated cognitive burden [21] grow explosively. Consequently, individuals, organizations and even entire communities cannot have more than an infinitesimal understanding of all these potential combinations. Actors must focus and recombine locally from a limited set of components and combinations, as too much diversity diminishes the likelihood that sensible meaning can be attached to novelty [69, 76]. This implies the need for a stable or dominant subcomponent configuration that enables sense-making and provides both positive heuristics that determine where to search, as well as negative heuristics that specify where not to search [7].

Conversely, before a dominant subcomponent configuration or sense-making structure does emerge, stakeholders need to literally consider all potential (re-)combinations, as they can all become the basis of the future dominant design. Increasing the diversity of the knowledge base in this (seed) stage actually diminishes possibilities for sense-making and absorption, and yields high integration costs. Hence, in the seed stage of component development, antecedent diversity results in a fragmentation of resources and a duplication of research efforts. This logic provides:

**Hypothesis 4:** Antecedent diversity is negatively (positively) associated with component growth in the seed (growth) stage of development.

### Descendant diversity

The technological descendants of the focal niche can be seen as the technological extensions of the component’s technology, or its application domain in the broader technological environment or landscape. The more diverse its application domain, the more the component’s technology is diffused, and the more attractive the component technology becomes for potential adopters [66]. After all, technologies develop as they diffuse, and, as they progress, they become more attractive for potential adopters [77], offering useful and sufficient ‘feedstock’ for subsequent descendant technologies. Obviously, increasing the attractiveness of the technological component enhances its subsequent growth. We therefore formulate:

**Hypothesis 5:** Descendant diversity is positively associated with component growth.

## Data and methodology

Within innovation and technology studies, there is a long history of using patent data [78, 79, 80]. The reason is that patent statistics provide an extremely useful data source, given their coverage, transparency and accessibility [81]. Therefore, patent data are considered by many as the most direct, detailed and objective measures of innovation [82, 83, 84]. In the words of Zvi Griliches [80], who was one of the first to study technological change empirically, “in this desert of data, patent statistics loom up as a mirage of wonderful plentitude and objectivity.” Especially within biotechnology, patent statistics are a good indicator of the evolution of technology [85, 86, 87].

For many ‘dedicated biotechnology firms’, or DBFs, a common strategy is to patent and subsequently license out or sell their technological knowledge. Large pharmaceutical firms also use patents strategically–for example, as leverage or bargaining chips in negotiations, or to stifle developments by competitors. Since the Bayh-Doyle act–which allows the patenting of research findings funded by means of federal grants–research institutes, such as universities, are also highly active to patent their newly discovered technology. As a result, all landmark innovations within biotechnology have been patented. Many scholars therefore believe that patent data are well suited to delineate different stages of technological evolution and the characteristics of the technological (selection) environment in this domain. Although we acknowledge that patent data only represent the explicit portion of technological knowledge contained within an organization or industry, due to the importance of patents within biotechnology, it is also a fair proxy for the tacit portion of technological knowledge. Especially within biotechnology, patents form a reliable indicator of technological developments [85, 86, 87]. As such, biotechnology is also ideally suited for our current purposes. Moreover, biotechnology is also almost purely science based, which is considered by several authors as a defining characteristic of emerging technology [44, 88]. We have to note that this fact could potentially limit the generalizability of our model to other science-based technologies.

We use patent data from the United States Patent and Trademark Office (USPTO), as this is the most complete dataset for technology analysis [61, 89]. Because the US is the largest marketplace for biotechnology, it is standard practice for non-US organizations to patent in the US [90]. Additionally, because of the tight linkage between biotechnology and science, biotechnology is a relatively autonomous technology that does not primarily depend on developments within other technologies. This makes biotechnology the ideal setting for an empirical analysis of the kind that we are proposing here. Within the USPTO, biotechnology is represented by patent classes 435 and 800. We define our technological components at the primary sub-class level of biotechnology, resulting in a total of 27 primary sub-classes (i.e., 18 primary sub-classes in class 435, and 9 primary sub-classes in class 800). The classification system at the USPTO actually distinguishes between 15 hierarchical levels, and classifies patents into one of these levels depending upon the level of detail of the patent. Our primary sub-classes refer to the highest hierarchical level. In total, biotechnology is composed of 750 sub-classes that are distributed among 8 hierarchical levels.

### Measures

Component growth, our dependent variable, is a count of the number of patents that enter a focal technological component in a particular month from January 1976 until December 2003. Because we have repeated observations for the same components, our data constitute a cross-sectional time-series or panel data structure. This panel is unbalanced, though, as not all components were in existence at the start of our time window.

Regarding our density measures, System density is a count of the total number of patents (in thousands) contained within the whole domain of biotechnology in the month prior to our dependent variable. To avoid double counting, we have subtracted focal component density. Next, focal Component density is a count of the total number of patents (in thousands) in the focal component in the month prior to the dependent variable. This measure represents the stock of patents contained in the focal component at any given time. Finally, Organizational density is a count of the number of organizations active in the component in the previous twelve months in thousands.

Component diversity represents the extent to which developments take place in different subcomponents, and is measured by the distribution of patents across all USPTO sub-classes over the previous twelve months. To measure component diversity, we use Shannon’s [91] diversity measure, which implies

Dit=∑j=1j=JPijtln⁡(1pijt) (1)

where *D*_*it*_ denotes to the diversity of component *i* at time *t*, and *P*_*ijt*_ is the share of patents in subcomponent *j* at time *t* in component *i*, time *t* refers to the twelve-month period prior to the month of observation of our dependent variable, and *J* denotes the number of subcomponents associated with the component.

The component’s Antecedent diversity is calculated over the previous twelve months according to (1), but now *P*_*ijt*_ refers to the share of citations made from focal component *j* to antecedent component *i* at time *t*. Correspondingly, the component's Descendant diversity is calculated over the previous twelve months using (1), where *P*_*ijt*_ is the share of citations received from descendant component *i* to focal component *j* at time *t*.

We also add a number of control variables. First, we include the number of previous entries and its square–Previous entry and Previous entry^2^ –to allow for the estimation of dynamic models [92]. This pair of measures effectively controls for the (un)favorable conditions within the environment that may (dis)encourage component entry [62, 93]. We also include a number of Year dummies in all our analyses to control for year-specific effects. More specifically, we enter year dummies for the years 1999 until 2003, because previous analyses have indicated that these years are characterized by significantly lower entry rates. We have also run our analyses with the full set of year dummies, which leads to both quantitatively and qualitatively similar results (available upon request). Descriptive statistics of the variables are provided in Table 2, our correlation matrix is given in Table 3, and a description of symbols used is presented in Table 4.

**Table 2:** Descriptive statistics.

Variable	mean	S.D.	min	max	25th %	50th %	75th %
Component growth	5.017	14.354	0.000	217.000	0.000	1.000	4.000
Previous entry	0.005	0.014	0.000	0.217	0.000	0.001	0.004
System density	16.554	11.166	2.879	44.954	7.701	12.551	22.606
Component density	0.669	1.628	0.001	15.139	0.022	0.085	0.571
Organizational density	0.034	0.077	0.000	0.666	0.001	0.008	0.029
Component diversity	1.827	1.496	0.000	4.706	0.000	1.931	3.172
Antecedent diversity	1.794	1.208	0.000	4.270	0.684	2.040	2.790
Descendant diversity	1.823	1.055	0.000	3.940	1.070	2.060	2.660

**Table 3:** Correlation matrix.

	Variable	1	2	3	4	5	8	9	10
1	Component entry	1.00							
2	Previous entry	0.93	1.00						
3	System density	0.11	0.12	1.00					
4	Component density	0.88	0.88	0.10	1.00				
5	Organizational density	0.94	0.94	0.15	0.95	1.00			
8	Component diversity	0.38	0.38	-0.08	0.48	0.46	1.00		
9	Antecedent diversity	0.34	0.34	0.30	0.39	0.42	0.63	1.00	
10	Descendant diversity	0.29	0.29	0.34	0.36	0.37	0.55	0.84	1.00

**Table 4:** Description of symbols.

Symbol	Description
*C*	Component density
*D*_*it*_	Diversity of component *i* at time *t*
*i*	Component
*j*	Subcomponent
*LL Base*	Log likelihood of the base model
*LL ML*	Log likelihood of the multi-level model
*O*	Organizational density
*P*_*ijt*_	Share of patents in subcomponent *j* of component *i* at time *t*
*S*_*s*_	System density in the seed stage of component development
*S*_*g*_	System density in the growth stage of component development
*t*	Time
*Y*	Component growth
*χ*_*2*_	Chi square value of multi-level model

As can be seen in Table 3, our density measures are highly correlated with one another, which means that we must proceed with caution to prevent that our findings are the result of multicollinearity. Theoretically, multicollinearity is not really an issue, as our theory needs such a special model. Indeed, in most empirical studies in the organizational ecology tradition, multicollinearity issues have to give way to what is required by theory. For example, to test the famous density dependence theory, density and density squared have to be entered in the same model. The near-perfect multicollinearity in this type of models that emerges as an inevitable result does not undermine these models’ value added.

### Estimation

To distinguish between different stages of component evolution, we employ a structural break model. As the name implies, a structural break model allows an investigation of whether a time series features a structural break, which means that there is a significant change in the effects of the model between two (or more) periods [92]. The general structural break model captures a change both in the intercept and in the slope of one or more variables. However, because we do not expect a sudden change in the intercept to occur between different stages, we do not need to allow for a change in both intercept and coefficient values. Instead, we expect a change only in the effect of system density on component growth. Our structural break model can thus be specified as

$$Y=\left[\begin{array}{cc} S_{s} & \begin{array}{cc} 0 & X_{s} \end{array}\\ 0 & \begin{array}{cc} S_{g} & X_{g} \end{array} \end{array}\right]\beta +\varepsilon$$ (2)

where *Y* refers to our dependent variable (i.e., component growth), *S*_*s*_ to system density in the seed stage, *S*_*g*_ to system density in the growth stage, *X* to variables whose coefficient values are not expected to change, *β* to the coefficient vector, and *ε* to the associated error term.

By estimating this structural break model for each of our technological components individually, we are able to determine at which point in time system density has a significant positive effect on component entry that marks the beginning of the growth stage of our components. The dependent variable for this model is component growth, as reflected by the entry of inventions into our components, which is a count variable. The baseline model for analyzing count data is the Poisson distribution. After adding covariates to the distribution, this gives the Poisson regression model. However, the restriction of applying the Poisson distribution in linear regression analysis is that, after adding covariates, the sample mean and conditional variance of the dependent variable have to be equal [94]. Because our data suffer from overdispersion, we accommodate for this by adding a dispersion parameter δ to the Poisson regression model. This results in a so-called negative binomial regression model (i.e., NB2 in [94]), and is defined as

$$\mathrm{Pr}\left(y_{t}|x_{t},\delta_{t}\right)=\frac{e^{-\lambda_{t}\delta_{t}}{\left(\lambda_{t}\delta_{t}\right)}^{y_{t}}}{y_{t}!}$$ (3)

where *λ*_*it*_ is the deterministic function of covariates.

Our main covariate is system density, which we split on a yearly basis into a before (i.e., seed) and after (i.e., growth) part to determine the location of the structural break, if present. For example, for the year 1980, we distinguish between system density before 1/1/1980 (i.e., 1976–1979) and system density after 1/1/1980 (i.e., 1980–2003), to then investigate the coefficient values of the before and after part of system density. We accept the structural break as the distinction between the seed and growth stage if (1) we find a non-significant effect for ‘before system density’–that is, system density in the seed stage of development–and (2) we find a significant positive effect for ‘after system density’–that is, system density in the growth stage of development. In the case of multiple valid options, we select the breakpoint with the higher Log-Likelihood value (i.e., the best fitting model).

We add several controls. First, we include the previous number of entries and its square as a control for favorable conditions [62, 93]. We use a dynamic regression model to control for unobserved heterogeneity or serial correlation. Moreover, we control for organizational and component density using the Generalized Yule specification, which means that we include the logarithmic and quadratic term of both organizational and component density as controls [95]. Because of the high correlation between our density measures, we have built up our density dependent models incrementally through stepwise regressions using both the Generalized-Yule and the Log-Quadratic specification (available upon request) of density dependence in our analyses. These analyses indicate that our estimates are highly stable and not the result of multicollinearity. We report the Generalized-Yule specification as this better connects to the theory [95]. This implies

$$y_{t}^{\prime }=\beta O_{t}∙\omega C_{t}∙\exp\left(\alpha +C_{t}^{2}+\rho O_{t}^{2}+\varsigma S_{st}+\upsilon S_{gt}\right)∙\exp\left(\varepsilon_{t}\right)=y_{t}\delta_{t}$$ (4)

where *O*_*t*_ is organizational density at time *t*, *C*_*t*_ is component density at time *t*, *S*_*st*_ is system density in the seed stage of development at time *t*, and *S*_*gt*_ is system density in the growth stage of development at time *t*.

After delineating the different stages of component development, we can continue to test our hypotheses. Our data involves monthly observations from 1976 to 2003 for 27 technological components, so we are dealing with a cross-sectional time-series or panel data structure. When modeling panel data, there are basically two options: employing a dynamic regression model or a panel regression model. We already apply a dynamic regression model because we include the previous occurrences of our dependent variable (i.e., previous entry) in our analysis. We also employ a panel regression model, which comes in two basic flavors: (1) a fixed effects model, which adds a dummy per panel, and so effectively removes all variance between panels–also referred to as a within-variance model; and (2) a random effects model, which assumes that heterogeneity is randomly distributed and therefore makes it possible to utilize both the within- and between-panel variance. More specifically, according to Hausman, Hall, and Griliches [96], the random effects negative binomial model allows the variance of the effects to differ in the within and between dimensions, and is essentially a ‘variance components’ version of the negative binomial. When this random effect is drawn from Gamma distribution, mixing this Gamma distribution with the Poisson distribution (i.e., the baseline to model count data) effectively creates a Beta distribution, with two parameters (i.e., *r* and *s*). This yields the model

$$\mathrm{Pr}\left(Y_{it}=y_{it}|x_{it},\delta_{i}\right)=\frac{\Gamma (\lambda_{it}+y_{it})}{\Gamma (\lambda_{it})\Gamma (y_{it}+1)}{\left(\frac{1}{1+\delta_{i}}\right)}^{\lambda_{it}}{\left(\frac{\delta_{i}}{1+\delta_{i}}\right)}^{y_{it}}$$ (5)

with

$$\lambda_{it}=exp(x_{it}\beta +{offset}_{it})$$ (6)

In the random effects overdispersion model, *δ*_*i*_ is allowed to vary randomly across groups, and 1/(1+ *δ*_*i*_) ~ Beta(*r*,*s*). As the name already indicates, the random effects model has the limiting assumption that the unobserved heterogeneity is randomly distributed, and therefore independent from the regressors (we have used the XTNBREG, FE command in Stata 8 to estimate our fixed effects model, and the XTNBREG, RE command in Stata 8 to estimate our random effects model). To determine whether this is indeed the case, we apply Hausman’s [97] specification test (i.e., the HAUSMAN command in Stata 8), which tests whether the coefficients from the consistent (i.e., the fixed effect) model are similar to the coefficients from the efficient (i.e., the random effects) model. In other words, Hausman’s specification test allows us to confirm that there is no correlation between our predictor variables and the error term, and that our model is specified correctly (a misspecification would lead to biased coefficients).

Our data involve left-censoring, as data are missing for the beginning of the history of our population (patent citations are missing for the pre-1976 period). However, this does not imply a survivor bias, as we have all cohorts. Furthermore, while scientific developments in biotechnology were surely stimulated after the discovery of DNA in 1972, commercial activity only took off after 1980 [98]. Furthermore, we have data on a cross-section of different technologies within biotechnology, implying that several new and emerging technologies are represented.

## Results

In Table 5, we provide the estimates of our structural break model on the basis of the effect of system density on component entry. As can be seen from this table, 13 out of a total of 27 components display a structural break, and thus enter the growth stage during our window of observation. All of these 13 components demonstrate significant model improvements when using the suggested structural break point. The improvement in model fit can be determined by comparing two times the difference in the Log-Likelihood to a *χ*^*2*^ distribution with one degree of freedom (column 8 of Table 5). Even though component 800001 does not show a significant coefficient value for community density in the growth stage of development (i.e., *S*_*g*_ in Table 5), we do use the time of the break in our subsequent analysis. The reason is that (i) the coefficient value is consistently positive after its structural break point and (ii) the model improvement is significant (i.e., *p* < .05).

**Table 5:** Negative binomial regression estimates of multi-level structural break model of biotechnology’s components.

Component	*S*	LL Base	Growth	*S*_*s*_	*S*_*g*_	LL ML	*χ*^*2*^
435001^†^	0.271***	-298.91	1/1987	0.100	0.292***	-292.75	12.32***
435002	0.464***	-368.42	1/1976	n.a.	n.a.	n.a.	n.a.
435003	-0.053	-137.24	n.a.	n.a.	n.a.	n.a.	n.a.
435004^†^	0.063	-1,202.77	1/1979	0.067	0.142***	-1,199.34	6.86***
435005	0.385***	-1,123.33	1/1976	n.a.	n.a.	n.a.	n.a.
435006^†^	0.098***	-482.18	1/1981	-0.167	0.130***	-478.74	6.88***
435007^†^	0.181***	-216.34	1/1986	0.063	0.246***	-213.91	4.86**
435008	-0.083**	-553.06	n.a.	n.a.	n.a.	n.a.	n.a.
435009	0.295***	-868.42	1/1976	n.a.	n.a.	n.a.	n.a.
435010	0.305***	-437.80	1/1976	n.a.	n.a.	n.a.	n.a.
435011^†^	0.117	-670.91	1/1984	0.109	0.189***	-668.58	4.66**
435012^†^	0.134**	-356.59	1/1984	0.029	0.185***	-354.16	4.86**
435013	0.033	-46.67	n.a.	n.a.	n.a.	n.a.	n.a.
435014	0.002	-743.54	n.a.	n.a.	n.a.	n.a.	n.a.
435015	0.214***	-420.27	1/1984	n.a.	n.a.	n.a.	n.a.
435016	0.081	-583.04	1/1976	n.a.	n.a.	n.a.	n.a.
435017	-0.013	-751.86	n.a.	n.a.	n.a.	n.a.	n.a.
435018^†^	0.485***	-55.19	1/1990	0.169	0.438***	-52.63	5.12**
800001^†^	0.347	-116.09	1/1996	-0.769	0.101	-113.17	5.84**
800002	0.848***	-78.29	1/1991	n.a.	n.a.	n.a.	n.a.
800003^†^	0.290***	-211.29	1/1992	-0.818	0.295***	-208.82	4.94**
800004^†^	0.180***	-124.32	1/1991	0.022	0.380***	-118.3	12.04***
800005^†^	0.099*	-402.28	1/1996	0.062	0.902**	-398.92	6.72***
800006^†^	1.193***	-40.97	1/1988	-0.592	1.067***	-39.2	3.54*
800007	0.051	-28.99	n.a.	n.a.	n.a.	n.a.	n.a.
800008	0.060	-363.91	n.a.	n.a.	n.a.	n.a.	n.a.
800009^†^	0.083	-471.99	1/1986	-0.082	0.162**	-468.06	7.86***

* significant at 10%.

** significant at 5%.

*** significant at 1%.

^†^ Component experiences a structural break during our window of observation; standard errors in brackets.

*S* = system density. *S*_*s*_ = system density in the seed stage of development. *S*_*g*_ = system density in the growth stage of development. LL = Log likelihood. Base = comparison model. ML = multi-level structural break model. Growth = month of start growth stage component. *χ*^*2*^ = Chi square value of multi-level model = -2 * (LL Base–LL ML).

For the remaining components, we did not find a structural break on the basis of our criteria. This implies that these components are in the same stage of development for the whole period of observation. The question then becomes whether they are in the seed or growth stage of development during our time window. Again, on the basis of the effect of system density on component entry, we can determine the appropriate stage. As can be seen in Table 5, components 435002, 435005, 435009, 435010, 435015, 435016 and 800002 are assumed to be in the growth stage from the start of our observation period. With the exception of component 435016, the effect of system density on component growth is significantly positive. The reason that we assume that component 435016 is in the growth stage of development is that (1) the effect of system density on component growth is consistently positive, (2) the mean of component entry is 2.74, which is the 10^th^ highest of all components, and (3) maximum component entry is 14 components per month, which is 12^th^ highest of all components. The other components (i.e., components 435003, 435008, 435013, 435014, 435017, 800007, and 800008) are assumed to be in the seed or formative stage during the observation period. To determine the performance of our multi-level structural break model, we have also estimated different stages of component development using a Bass [35] model and a random assignment model. We have compared the quality of the alternative distinctions between the different stages of development using negative binomial panel regression models. Our multi-level structural break model performs significantly better (results available on request).

To determine whether we are indeed observing different stages of technological development for our components, we first have a look at the confidence interval of our dependent variable (i.e., component entry). As can be seen in Table 6, the mean count of patents that enter our technological components (entry) in the growth stage is significantly higher than in the seed stage of development. However, this is rather obvious because the seed stage is temporally positioned before the growth stage and the entry of inventions increases over time. So, this does not say too much about the quality of the structural break according to our multi-level model. However, as can be seen in Table 6, the growth rate (i.e., growth = entry/density) is also significantly higher in the growth stage. So, it appears that the distinction between the different stages of technological development is a substantive rather than a spurious one. Therefore, we proceed with this distinction in our subsequent analysis. More specifically, we estimate two negative binomial random effects panel models, namely one for the seed period and one for the growth period, to prevent possible multicollinearity issues that would result from a structural break model.

**Table 6:** Confidence interval for entry and growth in different stages of technological development.

Variable	Obs	Mean	S.E.	95% confidence interval	
Growth (seed)	2996	0.0082	0.0008	0.0067	0.0097
Growth (growth)	5052	0.0131	0.0008	0.0115	0.0146
Entry (seed)	2996	1.8435	0.0623	1.7214	1.9655
Entry (growth)	5052	6.8804	0.2481	6.3941	7.3668

We have performed Hausman’s specification test, revealing that a random effects specification is indeed appropriate, as our independent variables are not correlated with the random disturbance term.

Table 7 presents the estimates for the random-effects negative binomial dispersion model of patent counts in the seed and growth stage of component development that we use to evaluate our hypotheses.

**Table 7:** Negative binomial dynamic multi-level panel regression estimates of the seed and growth stage of technological evolution.

	Seed stage	Growth stage
Previous entry	24.035*	7.376***
	(-13.895)	(-1.198)
Previous entry^2^	-599.037	-20.217
	(-666.034)	(-5.454)
LN(Organizational density*1000)	0.538***	0.445***
	(-0.078)	(-0.047)
Organizational density^2^	0.000	0.000
	(0.000)	(0.000)
System density	0.092	0.066***
	(-0.082)	(-0.023)
System density^2^	-0.000	-0.000
	(-0.001)	(-0.000)
LN(Component density*1000)	0.290***	0.283***
	(-0.070)	(-0.041)
Component density^2^	0.000	0.000
	(0.000)	(0.000)
Component diversity	0.446***	-0.151***
	(-0.107)	(-0.049)
Antecedent diversity	-0.300***	0.033
	(-0.068)	(-0.037)
Descendant diversity	0.200***	0.164***
	(-0.075)	(-0.046)
Constant	-4.768***	-3.795
	(-1.457)	(-0.553)
Observations	2976	5045
Number of components	20	20
Degrees of freedom	29	29
R	3.655***	1.880***
S	2.291***	1.216***
LL Constant	-3,584	-10,413
LL Comparison	-3,223	-8,842
LL Full model	-3,051	-8,419
Pseudo R^2^	0.149	0.191

* significant at 10%.

** significant at 5%.

*** significant at 1%.

Standard errors in brackets.

Hypothesis 1 posits that, following density dependence logic, component density is positively associated with component growth in both stages of technological development, and that this effect is not significantly weaker in the growth stage of development. We find strong support for this hypothesis. In the seed (growth) stage of development, moving niche density from its 1^st^ quartile to its 3^rd^ quartile increases niche entry with 216% (136%). At first sight, it might appear that component density has a different effect in the different stages of component development. However, this is mainly due to the different value ranges of component density in the different stages of development. To determine whether the effect of density is significantly weaker in the growth stage of development, we have estimated a structural break model following (2) where component density was split into its seed and growth part. According to this analysis, the effect of component density is not significantly lower in the growth stage of development, thus providing support for our hypothesis.

Hypothesis 2 implies the assumption that the number of organizations that adopt the component technology is a proxy for the legitimation of technology. Because legitimation processes are more important in the formative (seed) stage of development, a stronger positive effect is expected in this stage. Our estimates provide some, but not full support for this hypothesis. We do find a stronger effect for the linear term of organizational density in the seed stage of development, which means that the effect of organizational density is stronger in the seed stage than in the growth stage. However, additional analyses (not reported here, for the sake of brevity; available upon request) do not reveal a significant difference in the two functions. Even though the coefficients are not significantly different, the difference in effects is rather large. Moving organizational density from the 1^st^ to the 2^nd^ quartile in the growth stage of development, increases component growth with 71%; moving organizational density from its median value to the 3^rd^ quartile, further increases component growth with 85% (236%) in the growth (seed) stage of development.

Hypothesis 3 argues that niche diversity is positively (negatively) associated with component entry in the seed (growth) stage of development. This hypothesis is fully confirmed by our estimates. In the seed (growth) stage of development, a standard deviation increase in niche diversity increases (decreases) niche entry with 95% (20%).

Hypothesis 4 states that antecedent diversity is negatively (positively) associated with component entry in the seed (growth) stage of technological development. We only find partial support for this hypothesis. On the one hand, although the effect of antecedent diversity is positive in the growth stage of development, this effect is non-significant. On the other hand, antecedent diversity does have a significant negative effect on component growth in the seed stage, and increasing antecedent diversity with one standard deviation decreases component entry with 34%. Additional analyses (available upon request) also reveal that the coefficients of antecedent diversity are significantly different in the two stages of development.

Finally, Hypothesis 5, which postulates that descendant diversity has a positive effect in both stages of development, can also be confirmed by our estimates. Increasing the value of descendant diversity with one standard deviation in the seed (growth) stage of development, increases niche entry with 27% (16%). Further analysis indicates that there is no significant difference in the coefficient value for descendant diversity in the two stages of development.

## Discussion and conclusion

### Contribution

Overall, our estimates generate broad support for many of our hypotheses, providing evidence for our claim that there are indeed different stages of technological evolution at the component level, each associated with specific stage-dependent evolutionary processes. This allows us to move beyond a mere caricature of technological evolution as an S-shaped growth pattern [99], and enables an investigation into the processes that underlie this characteristic growth pattern. Our estimates reveal an intricate, but characteristic pattern of technological evolution, with different processes operating at different stages of development and at different levels of analysis. Obviously, this is not a surprising observation, as it is well documented that technological change is a highly complex, dynamic, and inherently multi-level phenomenon [10]. Although it is impossible to draw strong conclusions and implications on the basis of a single study, our pattern of significant findings does demonstrate that further investigation is certainly warranted–not only to further refine our theory, but also to validate our findings in other settings (e.g., for non-emerging technologies).

By conceiving of technology as a system composed of a set of interdependent components that evolve through different stages of technological development, we have effectively created a multi-level and systemic evolutionary model of technological growth. According to our model, when a component develops a stable role within a technological system (i.e., after a dominant subcomponent configuration is established), it begins to form an integral part of that system, and the processes that direct the component’s growth and evolution change. On the one hand, before the emergence of this dominant subcomponent configuration (i.e., in the seed stage of development), focal diversity has a positive effect on component growth. Hence, alternative subcomponent configurations attract resources and attention to further the development of this array of alternative technological structures (or technological options). However, despite the advantageous effect of alternative technological structures (i.e., subcomponent configurations), these alternative configurations should not be based upon diverse technological knowledge, which is indicated by the negative effect of antecedent diversity in this stage. That is, due to the lack of a dominant subcomponent design, diverse knowledge reduces the sense-making capabilities and increases integration costs.

On the other hand, after a dominant design has been established, the component is legitimated at the system level, which means that it now forms an integral part of the system’s structure. This means that alternative subcomponent configurations (i.e., indicated by component diversity) thwart resources and attention from the agreed-upon dominant subcomponent configuration, which hampers technological growth. Moreover, because the dominant design provides a means to make sense of the environment, knowledge-base diversity no longer has a negative effect on technological growth. The dominant subcomponent configuration (i.e., a dominant design) acts as some sort of filter or heuristic, which not only redirects resources and attention in the technological environment, but also acts as a sense-making structure with which to interpret the environment. Hence, stability is needed to make sense of the world, hereby reducing uncertainty, enabling specialization, and facilitating cumulative changes.

When taking this previous argument to a higher level of aggregation, after each component is identified and has established a stable role within the technological system, the system itself becomes a stable and predictable integrated whole, and enters the growth stage of development. That is, the system has formed a dominant component configuration, or a dominant system design. In other words, stability travels upward. This connects to Barley’s [100] finding that technology-induced micro-social dynamics travel upwards in an orderly manner. By explicating how this stability develops from the bottom up, it is possible to elucidate the emergence of structures at higher levels of analysis. Furthermore, the model proposed here can be easily extended to include multiple levels of analysis. Then, it can allow for fine-grained analyses of technological systems and subsystems. For example, it is possible to conceive inventions bundled into components, components bundled into products and processes, and products and processes bundled into paradigms. Within such a hierarchically nested multi-level model, levels are nested within one another, and wholes are composed of elements at lower levels, which are themselves part of more extensive wholes [101]. Indeed, more and more work recognizes the value added of paying explicit attention to the nested nature of multiple levels of analysis [102]. By investigating the formation of these stable configurations or structures at multiple levels of analysis, we can develop greater insight into the path-dependent nature of technology, and draw important managerial and policy implications. Obviously, our work here implies only an initial first step, and much work needs to be done to develop a solid foundation for the development of such a hierarchically nested, multi-level model of technology.

The current literature on technology and innovation treats the origins of novelty as exogenous and random, and focuses mainly on processes of diffusion and absorption [20, 103]. In contrast, in the current study, by distinguishing between the seed and growth stage of development, separated by a dominant design, we focus on both the process of knowledge creation and the process of knowledge diffusion. On the one hand, the seed stage of development can be characterized by the (social) construction of a dominant subcomponent design, in which the basic configuration of the technological component is created. On the other hand, in the growth stage of technological development, a dominant component design exists that outlines the basic configuration of the component and directs its future growth, development and evolution. So, the growth stage can be characterized as technologically deterministic, where the dominant subcomponent design of the technological component diffuses throughout the environment, and the stakeholders in the environment adjust their structures and procedures to facilitate the development of the agreed upon dominant subcomponent design configuration of the technological component. Our systemic multi-level evolutionary model thus enables an analysis of both the process of knowledge creation and the subsequent diffusion of the created knowledge.

By taking into account the lineage of technology in combination with diversity, we found that the embeddedness of a component (i.e., how it relates to other technological components) has a substantial effect on its growth rate, illustrating the importance of a socialized perspective towards technological change. Both sides of a component’s technological lineage, namely the diversity of its antecedent and of its descendant technologies, have a strong effect on the focal component’s growth rate. As illustrated above, this has generated more insight into the twin processes of knowledge creation and diffusion, characterized by the different stages of technological development. Even though we have demonstrated the significance of the diversity of these dimensions of the technological component, it is also possible to conceive of other characteristics of these dimensions. For example, results from some preliminary analyses indeed indicate that antecedent and descendant stability also play an important role in the evolution and growth of technology. A thorough investigation of the effect of different characteristics of antecedent and descendant technology on technological development would surely contribute much to our understanding of the growth and evolution of technology.

### Future research

In this paper, we have found that an emerging technology’s components are characterized by two stages of development: i.e., the seed and growth stage. We have shown that the different levels of analysis are not independent from one another. More specifically, the growth of component technology not only influences the growth of the system as a whole (as the system is an aggregation of its individual components), but the configuration of components at the system level also influences subsequent component growth (i.e., components are legitimated at the system level). As such, our analysis stresses the crucial role of cross-fertilization at the level of underlying technological components and the existence of an intricate link between different levels of analysis. Actually, we believe that the S-shaped growth pattern characterizes technological populations at multiple levels of analysis, and that the different stages of the technology lifecycle (i.e., the seed, growth, maturity, and decline stage) at different levels of analysis are linked to one another. Obviously, future research is needed to really move deeper ‘inside the black box’ of technology development [18]. Some of the avenues that should be explored to peek inside this black box are as follows.

First, future research could explore how to draw the boundaries of technological populations at multiple levels of analysis. Ecological theories are typically silent about how boundaries should be drawn [12]. In this paper, we have used the classification system of the USPTO to identify the boundaries of our technological system and its component technologies. Clearly, this is not without its limitations, and there is no guarantee that this delineation of boundaries is indeed correct. Future research could explore alternative demarcations of technological systems and components. For example, the pattern of patent citations between technological components could be used to construct technological systems from the ground up. Such an analysis could also explore the fluidity of component and system boundaries in different stages of technological development. In the seed stage of technological development, stakeholders in the environment explore and experiment with alternative (sub-)component configurations in an effort to find the configuration that will become the basis for future cumulative growth and development, which implies a fluidity of component boundaries. Additionally, when considering the impact of general-purpose technologies (i.e., technologies that impact many other technologies), it would be highly informative to study the boundaries of different technological systems (i.e., the boundary between the general-purpose technology and the technologies on which it has an impact).

Second, to determine whether different levels of analysis are indeed characterized by the different stages of the technological lifecycle with intimate links between these stages across different levels of analysis would require studying technologies in their (near) final stages of development: i.e., mature technologies. For example, researchers could study the evolution of semiconductor technology, as this technology is mature and has penetrated much of our society.

Third, we implicitly assume that the seed stage of technological development is characterized by competition between alternative component configurations. However, in the current paper, we do not measure this competition in any way. Future research could explore whether processes of competition can indeed be witnessed between alternative component configurations. This implies the need of being able to identify different component configurations during the seed stage of system development. Although this is not an easy task, requiring an intimate understanding of the technological system that is being investigated, this would imply an important next step toward a deeper understanding of processes of technological development and evolution.

Fourth, we have established that technological populations are internally differentiated, and have explored how this internal differentiation influences technological growth and development by introducing insights from organizational ecology. An interesting avenue of future research could be to explore whether a further integration of organizational ecological insights would be beneficial. For example, researchers could more explicitly focus on how processes of natural selection affect the growth and evolution of technological populations. Using ecological frameworks, it is possible to study the competition and legitimation between technological populations at multiple levels of analysis. One interesting avenue would be to investigate the process of technological substitution: i.e., the replacement of one technology for another.

Fifth, to concentrate our attention on the evolution of technology, we have largely abstracted from the role of the organization. Obviously, the insights from this analysis should be integrated with extant knowledge about organizational evolution, first by means of theoretical exploration, and subsequently by means of empirical investigation. Here, we have opened the door to a plethora of highly interesting research questions that can be further developed and explored. More specifically, by providing a quantitative model that facilitates a distinction between different stages of technological evolution, it becomes possible to explore the consequence of these different stages for individual organizations, by examining the position of organizations within a technological component (or system) at different stages of development, and by relating this position to organizational performance and survival. At higher levels of analyses (i.e., at the level of an organizational population and community), we can relate the different stages of technological development to the processes of entry and exit of organizations and organizational forms. This would be an important contribution to the literature. After all, even though it is widely acknowledged that technology drives ecological processes, in population ecology, with few exceptions, connections between technological change and organizational evolution are not of central interest [104]. Moreover, a formal model of the evolution of technology would allow a systematic investigation into the co-evolution of technologies and organizations, a phenomenon that is recognized by many as being highly important [2, 8, 57, 104]. After all, even though technology is structured (mainly) by organizations (i.e., through the creation of a dominant design), technology subsequently structures organizations (i.e., after a dominant design has been established). Hence, technological growth is highly path dependent. So, technology not only liberates us, but also entraps us [105]. Hence, it is clear that technological change deserves a central role in any organization theory [10].

### Policy implications

There is a “considerable and growing interest in the emergence of novel technologies, especially from the policy-making perspective” [6]. The reason is that that emerging technologies have the ability to change the status quo [44, 88]. This has resulted in several policy initiatives, such as the”Future and Emerging Technologies” (FET) initiative funded by the European Commission in 2013 and the”Foresight and Understanding from Scientific Exposition” (FUSE) program funded by the US Intelligence Advanced Research Projects Activities (IARPA) in 2011 [6].

**Fig 2:**
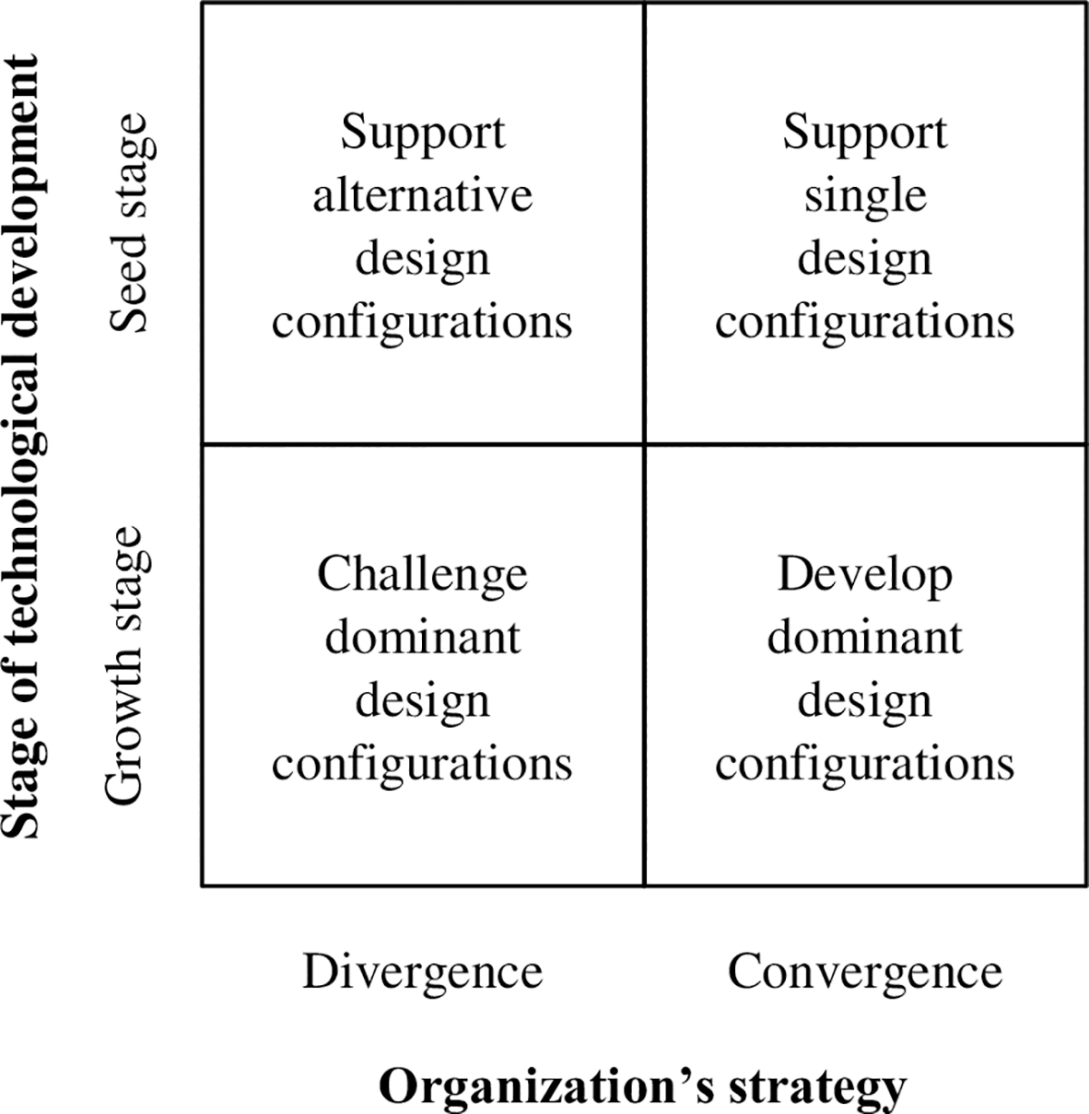
The organization’s strategy and stages of technological development.

Typically, policy-makers want to know which technologies to support. However, one of the biggest problems in technology selection is how to derive promising technology alternatives from blurred and diverging development directions that tend to characterize an emerging technology [106]. On the basis of our findings in this paper, we can provide some initial points of advice for policy-makers. The fact that diversity is positive in seed stages implies that policy should stimulate many alternatives in the initial stages of technological evolution. However, after the emergence of a dominant design, it is important to stop exploring and/or supporting alternatives, and instead focus on developing the dominant configuration that has been created. Hence, now, alternative programs could be terminated and resources could be redirected into developing the dominant technological design configuration (see Fig 2). At a lower level of analysis, policy-makers could use our model to identify which components are already legitimated at the system level, and therefore highly likely to become part of the stable design configuration. This way, instead of supporting broad technological developments, policy-makers can direct their resources to specific developments that are highly promising (i.e., the technology’s core components).

### Managerial implications

In firms, technology plays a highly important role and, on average, accounts for more than one-third of all business capital spending [106]. To remain competitive, technology-based firms need to rely on the renewal of existing technological resources and exploitation of new technologies [107]. To do so, firms are confronted with the difficult task to select which technological opportunities to pursue [108], and invest in those technological fields (and components) that provide a comparative (and competitive) advantage [106]. An important strategy is to actively pursue synergies and cross-fertilizations [40]. On the basis of our model, firms are able identify synergies and cross-fertilizations between technological components within a technological system.

Additionally, firms can also use the insights from Fig 2 to inform their technology strategy within technological systems. More specifically, in the seed stage of technological divergence, due to the existence of alternative design configurations, a firm has two options. First, it can support alternative design configurations to guarantee that the firm will have a stake in the future stable (or dominant) design configuration (i.e., applying a hedging strategy), whatever one will emerge as the winner. Second, the firm can also concentrate its attention by supporting one single design configurations under the expectation that this will become the future stable design configuration (i.e., applying a strategy of placing all eggs in one basket). In the growth stage of technological convergence, a stable design configuration does exist and the firm again has two options. First, it can work on alternative design configurations in an effort to overthrow the ‘current’ dominant design configuration. Alternatively, it can contribute to the development of (a part of) the dominant design configuration. Obviously, a mixture between strategies is also possible.
